# Knowledge, attitude and practice towards intestinal schistosomiasis among school-aged children and adults in Amhara Regional State, northwest Ethiopia. A cross-sectional study

**DOI:** 10.1186/s41182-024-00584-6

**Published:** 2024-03-11

**Authors:** Getaneh Alemu, Endalkachew Nibret, Arancha Amor, Abaineh Munshea, Melaku Anegagrie

**Affiliations:** 1 Department of Medical Laboratory Science, Bahir Dar University, Bahir Dar, Ethiopia; 2 Biology Department, Science College, Bahir Dar University, Bahir Dar, Ethiopia; 3 Mundo Sano Foundation and Institute of Health Carlos III, Madrid, Spain; 4 Health Biotechnology Division, Institute of Biotechnology (IoB), Bahir Dar University, Bahir Dar, Ethiopia

**Keywords:** Schistosomiasis, Knowledge, Attitude, Practice, Amhara, Ethiopia

## Abstract

**Background:**

*Schistosoma mansoni* causes intestinal schistosomiasis (SCH) in all regions of Ethiopia. Despite many years of mass treatment, the prevalence has not dropped significantly. The reduction of SCH transmission demands the integration of deworming with safe water, sanitation, and hygiene (WASH) activities. Adequate knowledge and a positive attitude towards SCH are critical to practicing those interventions. However, data on the knowledge, attitude, and practice (KAP) level in school and community settings is limited in Ethiopia.

**Methods:**

School and community-based cross-sectional studies were conducted from February to June 2023 among 634 school-aged children (SAC) and 558 adults. A pre-tested questionnaire was used to collect socio-demographic and KAP data. Records were entered and analyzed using SPSS software version 21. Correct responses for each of the KAP questions were scored as one, while incorrect or ‘I don’t know’ responses were scored as zero. Good knowledge, a positive attitude and good practice were declared if percentage scores were ≥ 80%, ≥ 90% and ≥ 75%, respectively.

**Results:**

Only 229 (19.2%) respondents, comprising 91 (14.4%) SAC and 138 (24.7%) adults, had ever heard of SCH. Adults, males, and urban residents had higher awareness level compared to their respective counterparts (p < 0.05). Only 28.4% of aware respondents knew that swimming or bathing in freshwater is a risk factor for schistosoma infection; 10.9% knew the etiologic agent; and 14.4% mentioned at least one sign and symptom associated with SCH. The majority (97.8%) of the respondents were willing to take therapeutic drugs, but only 37.6% believed that SCH is a serious disease. Regarding risky practices, 89.5% practiced swimming or bathing in freshwater, and 25.3% had no access to piped water. Among the aware respondents, only 18 (7.9%) had good knowledge, while 30 (13.1%) had a positive attitude towards SCH. Ninety-nine (43.2%) respondents had good *Schistosoma* infection prevention practices.

**Conclusions:**

The knowledge, attitude, and preventive practice level towards schistosomiasis are low in the study area. Therefore, strengthening school and community-based health education, along with mass drug administration (MDA), WASH, and a vector control program, is recommended for preventing SCH.

## Background

Schistosomiasis (SCH), or bilharzia, is a global public health problem caused by blood flukes of the genus *Schistosoma* [1, 2]. Globally, about 700 million people live in SCH-endemic areas, and more than 240 million are infected. Sub-Saharan Africa (SSA) carries more than 90% of the global cases, of which an estimated 280,000 people die every year [2, 3]. Ethiopia is one of the highly affected SSA countries, with about 53.3 million people at risk [4]. Of the two species of *Schistosoma* that are found in Ethiopia, *S. mansoni* is endemic to all administrative regions, while *S. haematobium* is limited to the eastern and western borders of the country [5, 6]. Amhara Regional State of Ethiopia, compared to other regional states, carries the highest burden of SCH, as evidenced by a pooled prevalence ranging from 17.5% to 41.1% in recent systematic reviews [7–10].

Transmission of SCH is linked to poor sanitary conditions and water-related activities like swimming or bathing in freshwater, use of surface water for in-house domestic activities, participation in agriculture, irrigation, and fishing [10, 11]. Participation in such activities exposes humans to the infection, which is initiated when the cercariae, the larval stage, penetrate the skin of people having contact with infested water [12, 13]. In the acute phase of the infection, *S. mansoni* causes cercarial dermatitis and serum sickness. As the infection progresses, especially in the chronic disease stage, common signs and symptoms such as abdominal discomfort, micro-ulceration, and superficial bleeding in the intestine, granuloma, hepatosplenomegally, and fibrosis are evident [14, 15]. Chronic *S. mansoni* infection in children in particular might result in anemia, growth retardation, cognitive impairment, and deficiency of important nutrients like vitamin A [16].

The risk of infection and morbidity due to *S. mansoni* is highest among school-aged children (SAC) due to behavioral and biological factors [10]. However, adults having frequent contact with freshwater during engagement in agriculture, irrigation, fishing, or other domestic activities are also at risk of *S. mansoni* infection. So far, health facility reports in Ethiopia have shown a higher *Schistosoma* infection rate in adults than in children [4].

Owing to its public health importance, the World Health Organization (WHO) recommended the implementation of selective mass drug administration (MDA), vector control, water sanitation and hygiene (WASH), and health education programs for the control and elimination of SCH [17]. The Federal Ministry of Health (FMoH) in Ethiopia has adopted the WHO recommendation and has been implementing it since 2012, but with more emphasis on MDA through the administration of praziquantel [4]. Despite satisfactory efficacy reports of praziquantel campaigns in the country, the prevalence of SCH did not drop significantly [18]. Continued re-infection is thought to be attributed to the community’s poor knowledge, attitude, and practice (KAP) about SCH [4, 19, 20]. Hence, integrated implementation of MDA, WASH, vector control programs, and community engagement are critical for the successful control and prevention of the infection. [21]. Although many studies have been conducted on the prevalence of SCH, data on the level of KAP in the population is very limited in Ethiopia, as a systematic search of studies revealed that only ten papers were published between 2006 and 2023 [unpublished systematic review]. Moreover, studies conducted in some regions of Ethiopia reported controversial findings [22, 23]. For instance, only 13% of the participants heard about the disease SCH in Benishangul Gumuz [22]. On the contrary, 94.4% of the study participants had good knowledge in western Tigray [23]. Hence, we expected that the KAP level in the Amhara region might still be low and have been contributing to persistent *Schistosoma* transmission in the region. However, updated data is required to prove this hypothesis and to recommend effective prevention and control of SCH in Amhara Regional State. Therefore, the present study aimed to assess the knowledge, attitude, and preventive practices towards intestinal schistosomiasis in school and community settings in Amhara Regional State.

## Methods

### Study design and study area

School and community-based cross-sectional studies in the respective school-aged children (SAC) and adults were conducted in selected districts of the Amhara Regional State from February to June 2023. The region is located at geographic coordinates of 8° 45′–13° 45′ N and 35° 46′–40° 25′ E. The mean annual temperature lies between 15 °C and 21 °C while the mean annual rainfall is about 1165 mm [24].

### Sample size calculation and sampling technique

The present study was conducted as part of an epidemiological survey of *S. mansoni* infection in Amhara Regional State using combined parasitological, immunological, and molecular diagnostic methods. Hence, a single population proportion formula was used to calculate sample size with the assumptions of *S. mansoni* prevalence of 50% in the study area and 5% margin of error (d = 0.05) at a 95% confidence level (Z α/2 = 1.96). A prevalence of 50% was considered because there was no similar study that assessed *S. mansoni* prevalence using combined diagnostic methods. As data were collected in different (school and community) settings, we calculated the sample size for SAC and adults separately, but with similar assumptions.

$${\text{Sample size for SAC}} = \frac{{\left( {{\text{Z}}\alpha /{2}} \right)^{{2}} {\text{P }}\left( {{1} - {\text{P}}} \right)}}{{{\text{d}}^{{2}} }} = \frac{{({1}.{96})^{{2}} \times 0.{5}\left( {{1}{-}0.{5}} \right)}}{{\left( {0.0{5}} \right)^{{2}} }} = {\text{384 participants}}$$

After adding 10% (39) for non-respondents and 1.5 for the design effect, the final sample size was 635. Districts in northwest Ethiopia were first grouped into four strata based on the SCH endemicity map generated in 2015 and updated in 2020 [4]. Then, 1–3 districts were randomly selected from each of the low, moderate, and high transmission strata based on the number of districts in each stratum. Two primary schools from each district were selected purposively based on their location near permanent water bodies (rivers, lakeshores, and irrigation canals). If more than two primary schools within a district were located near water bodies, two were selected randomly for data collection. Primary schools teaching at least up to grade eight were eligible for data collection. Once schools were identified, data on the number of students were obtained from all selected schools. Then, the sample size was proportionally allocated to each school and to each grade level. Finally, participants from each class were selected by a systematic random sampling technique using class rosters as a sampling frame.

Similar assumptions, as used for SAC, were taken to calculate the sample size for adults. Accordingly, a maximum of 635 adults were enrolled from seven districts. Individuals aged ≥ 18 years were considered adults for this study. One village located nearest to each data collection school was selected purposively. Households equal in number to the number of SAC recruited from the nearby school were selected by systematic random sampling using a list of households obtained from respective health posts. For this, we divided the number of households in the village by the number of households to be recruited to get the sampling interval (K). The first household was selected by lottery method, and every K^th^ household was included in the data collection. Then, one randomly selected adult in each household was recruited. If a house was found to be closed or no adult was present during two visits, the next household was considered for data collection. Volunteer SAC and adults who lived at least for the last six months in the current residence were included in the study.

### Data collection

A structured questionnaire adapted from previous similar studies was used for data collection [23, 25–28]. The questionnaire was prepared to have four sections. Section one contained questions about socio-demographic characteristics; section two contained knowledge assessment questions; section three contained attitude/perception assessment questions; and section four contained questions about SCH preventive practices. The questionnaires were prepared in English and translated to Amharic (local language) before administration. In the study area, schistosomiasis is known by the name ‘Bilharzia’ and snail intermediate host is known by its Amharic name ‘Qend Awta. So, we have used those local names during questionnaire-based data collection. The questionnaires were administered to those who could read and write, whereas questions in the questionnaire were read to those who could not read and write and their responses were recorded by trained nurses. For questions in sections two to four, correct responses were given a score of one, while incorrect responses were given a score of zero. Then results were summed, and a participant’s knowledge, attitude, and practice levels were determined based on Bloom’s modified cut-off value. Accordingly, scores of ≥ 80%, ≥ 90%, and ≥ 75% were interpreted as having good knowledge, a positive attitude, and good prevention practice, respectively [29].

### Data quality assurance

Data collectors received a two-day training to familiarize them with the data collection tools. The questionnaire was pre-tested in a non-selected school and nearby village to assess the reliability, validity, and comprehensiveness of each question. Then, it was updated based on findings of the pre-test before the actual data collection. The questionnaire was prepared in English, translated to Amharic (the local language), and re-translated back to English in order to ensure its consistency. The principal investigator checked the completeness of each questionnaire daily.

### Data analysis

Data were entered into an excel sheet 2010 and imported into SPSS version 21 (IBM SPSS Corp. Chicago, USA) for analysis. Socio-demographic characteristics and the KAP level of respondents were explained using descriptive statistics. Pearson’s chi-square test was computed at a 95% confidence level to determine an association between KAP level and socio-demographic factors. Associations between KAP and perceived factors were considered significant if p-value was ≤ 0.05.

## Results

### Socio-demographic characteristics of respondents

Out of 1270 (635 SAC and 635 adults) total sample size, 1192 (634 SAC and 558 adults) participated in the study, giving a response rate of 93.9%. The mean ± standard deviation (SD) among SAC participants was 11.02 ± 1.938, while the minimum and maximum ages were 6 and 14 years, respectively. The median age was 11. In adult participants, the mean ± SD age was 30.91 ± 10.524. The minimum, maximum, and median ages of adult participants were 18, 72, and 28 years, respectively. Among SAC participants, the majority (72.9%) were 10–14 years old while 60.4% of adult participants were 18–30 years old. Five hundred eighty-five (49.1%) participants were males, and 677 (56.8%) were rural dwellers (Table 1).

**Table 1:** Socio-demographic characteristics of school-aged children and adults in Amhara Regional State, northwest Ethiopia, February to June 2023 (N = 1192)

Variable	Category	Number (%)		
		SAC	Adults	Total
Age (in years)	6–9	172 (27.1)	–	172 (27.1)
	10–14	462 (72.9)	–	462 (72.9)
	18–30	–	337 (60.4)	337 (60.4)
	> 30	–	221 (39.6)	221 (39.6)
Gender	Male	336 (53.0)	249 (44.6)	585 (49.1)
	Female	298 (47.0)	309 (55.4)	607 (50.9)
Adults’ education	No formal education	–	236 (42.3)	236 (42.3)
	Primary	–	157 (28.1)	157 (28.1)
	Secondary	–	110 (19.7)	110 (19.7)
	College	–	55 (9.9)	55 (9.9)
Adults’ occupation	Farmer	–	202 (36.2)	202 (36.2)
	Day laborer	–	63 (11.3)	63 (11.3)
	Private business	–	138 (24.7)	138 (24.7)
	House wife	–	74 (13.2)	74 (13.2)
	Government	–	43 (7.7)	43 (7.7)
	Other		38 (6.9)	38 (6.9)
District	Bahir Dar Zuria	95 (15.0)	95 (17.0)	190 (15.9)
	Chagni	105 (16.6)	86 (15.4)	191 (16.0)
	Dera	111 (17.5)	107 (19.2)	218 (18.3)
	Jawi	67 (10.5)	56 (10.0)	123 (10.3)
	North Mecha	107 (16.9)	97 (17.4)	204 (17.1)
	Quara	61 (9.6)	31 (5.6)	92 (7.8)
	Tach Armachiho	88 (13.9)	86 (15.4)	174 (14.6)
Residence	Rural	345 (54.4)	332 (59.5)	677 (56.8)
	Urban	289 (45.6)	226 (40.5)	515 (43.2)
Family size	≤ 5	291 (45.9)	356 (63.8)	647 (54.3)
	> 5	343 (54.1)	202 (36.2)	545 (45.7)

### Knowledge about intestinal schistosomiasis

All participating SAC and adults were asked the question, ‘have you ever heard about schistosomiasis or bilharzia? Only 14.4% (91/634) SAC and 24.7% (138/558) adults responded ‘yes’. Accordingly, these respondents (91 SAC and 138 adults) were considered to have awareness about SCH. The main source of information about SCH were schools (65.9% for SAC, 67.4% for adults), followed by family or community (17.6% for SAC, 26.8% for adults). Among respondents who were aware of SCH, only 5.5% (5/91) of SAC and 14.5% (20/138) of adults said that *Schistosoma* infection is acquired by a worm. Similarly, only 1.1% (1/91) of SAC and 10.9% (15/138) of adults knew that snails are involved in the transmission of SCH. Regarding risky activities attributed to SCH transmission, 12.1% (11/91) and 1.1% (1/91) of SAC reported that defecating near water sources and swimming, bathing or playing near water bodies are risk factors for *Schistosoma* infection, respectively. Similarly, 15.2% (21/138) and 11.6% (16/138) of adults responded that defecating near water sources and swimming or bathing in water bodies were attributed to acquiring the infection, respectively. Among respondents who had awareness, 54.9% (50/91) of SAC and 60.9% (84/138) of adults said that SCH can be cured. The overall assessment of the participants’ knowledge level about the etiology, transmission, signs and symptoms, diagnosis, treatment, and prevention of SCH revealed that only 2.2% (2/91) of SAC and 11.6% (16/138) of adults had good knowledge (Table 2).

**Table 2:** Knowledge about schistosomiasis among school-aged children and adults in Amhara Regional State, northwest Ethiopia, February to June 2023

Knowledge Area	Responses	Number (%)		
		SAC	Adults	Total
Ever heard about SCH or bilharzia (SAC = 634, adults = 558)	Yes	91 (14.4)	138 (24.7)	229 (19.2)
	No	543 (85.6)	420 (75.3)	963 (80.8)
Source of information for SCH (SAC = 91, adult = 138)	School	60 (65.9)	93 (67.4)	153 (66.8)
	Family/community	16 (17.6)	37 (26.8)	53 (23.1)
	Health institutions/campaigns	7 (7.7)	26 (18.8)	33 (14.4)
	Media (radio/TV)	12 (13.2)	9 (6.5)	21 (9.2)
Causative agent of SCH	Worm	5 (5.5)	20 (14.5)	25 (10.9)
	Bacteria/virus/mosquito	0 (0.0)	2 (1.4)	2 (0.9)
	Don’t know	86 (94.5)	116(84.1)	202 (88.2)
Snails involvement in the transmission of SCH	Yes	1 (1.1)	15 (10.9)	16 (7.0)
	Don’t know	90 (98.9)	123 (89.1)	213 (93.0)
Activities attributed to SCH transmission	Defecating near fresh water	11 (12.1)	21 (15.2)	32 (14.0)
	Swimming/bathing or playing in fresh water	7 (7.7)	58 (42.0)	65 (28.4)
	Walking barefooted in water	1 (1.1)	10 (7.2)	11 (4.8)
	Don’t know	74 (81.3)	77 (55.8)	151 (65.9)
Means to know a person having SCH	By signs and symptoms	13 (14.3)	28 (20.3)	41 (17.9)
	By laboratory diagnosis	29 (31.9)	54 (39.1)	83 (36.2)
	Don’t know	61 (67.0)	82 (59.4)	143 (62.4)
Signs and symptoms of SCH	Abdominal pain	13 (14.3)	12 (8.7)	25 (10.9)
	Fever	1 (1.1)	4 (3.6)	5 (2.2)
	Bloody stool/diarrhea	–	5 (3.6)	5 (2.2)
	Nausea	–	3 (2.2)	3 (1.3)
	Cough	1 (1.1)	0 (0.0)	1 (0.4)
	Don’t know	78 (85.7)	118 (85.5)	196 (85.6)
SCH can be cured	Yes	50 (54.9)	84 (60.9)	134 (58.5)
	No	2 (2.2)	1 (0.7)	3 (1.3)
	Don’t know	39 (42.9)	53 (38.4)	92 (40.2)
Cure for SCH (SAC = 50, Adult = 84)	Modern medicine	50 (100.0)	83 (98.8)	133 (99.3)
	Traditional medicine	0 (0.0)	1 (1.2)	1 (0.7)
	Don’t know	0 (0.0)	1 (1.2)	1 (0.7)
SCH is preventable disease (SAC = 91, adult=138)	Yes	40 (44.0)	83 (60.1)	123 (53.7)
	No	0 (0.0)	0 (0.0)	0 (0.0)
	Don’t know	51 (56.0)	55 (39.9)	106 (46.3)
Method of SCH prevention (SAC = 40, Adult = 83)	Avoid contact with freshwater bodies	4 (10.0)	4 (4.8)	8 (6.5)
	Use clean water for drinking and washing	14 (35.0)	54 (65.1)	68 (55.3)
	Participate in mass treatment	4 (10.0)	6 (7.2)	10 (8.1)
	Other	17 (42.5)	11 (13.3)	28 (22.8)
	Don’t know	7 (17.5)	8 (9.6)	15 (12.2)
Knowledge level	Good	2 (2.2)	16 (11.6)	18 (7.9)
	Poor	89 (97.8)	122 (88.4)	211 (92.1)

### Factors associated with awareness for intestinal schistosomiasis

In the present study, a significantly higher proportion of adults were aware of SCH compared to SAC (24.7% vs. 14.4%, p < 0.001). Similarly, male respondents and urban residents had higher awareness levels (p < 0.05). By location, the highest and lowest awareness were from study participants in North Mecha (31.9%) and Dera (11.9%) districts, respectively (Table 3).

**Table 3:** Factors associated with awareness about schistosomiasis among school-aged children and adults in Amhara Regional State, northwest Ethiopia, February to June 2023

Variable	Category	No of respondents	No of persons with awareness (%)	χ^2^	p-value
Age group	School-aged child	634	91 (14.4)	20.595	< 0.001
	Adult	558	138 (24.7)		
Gender	Male	585	126 (21.5)	4.008	0.045
	Female	607	103 (17.0)		
Residence	Rural	677	116 (17.1)	4.355	0.037
	Urban	515	113 (21.9)		
District	Bahir Dar Zuria	190	36 (18.9)	33.100	< 0.001
	Chagni	191	40 (20.9)		
	Dera	218	26 (11.9)		
	Jawi	123	23 (18.7)		
	North Mecha	204	65 (31.9)		
	Quara	92	11 (12.0)		
	TachArmachiho	174	28 (16.1)		
SCH transmission setting of data collection sites	Low	612	127 (20.8)	2.280	0.320
	Moderate	406	74 (18.2)		
	High	174	28 (16.1)		
Family size	≤ 5	647	132 (20.4)	1.292	0.256
	> 5	545	97 (17.8)		

### Attitude towards intestinal schistosomiasis

Among a total of 229 (91 SAC and 138 adults) respondents who had awareness, 51.1% (117/229) thought that SCH is a preventable disease, while 47.2% (108/229) were not sure whether it is preventable or not. Only three SAC and one adult said that SCH is not a preventable disease. About 24.2% (22/91) of SAC and 46.4% (64/138) of adults believed that schistosomiasis is a serious disease. Only 13.1% of the respondents (4.4% SAC and 18.8% adults) had a positive attitude towards SCH (Table 4).

**Table 4:** Attitude towards schistosomiasis among school-aged children and adults in Amhara Regional State, northwest Ethiopia, February to June 2023

Attitude	Response	Number (%)		
		SAC	Adult	Total
SCH can be prevented (SAC = 91, Adult = 138)	Yes	40 (44.0)	77 (55.8)	117 (51.1)
	No	3 (3.3)	1 (0.7)	4 (1.7)
	Not sure	48 (52.7)	60 (43.5)	108 (47.2)
SCH is prevalent in your area	Yes	4 (4.4)	34 (24.6)	38 (16.6)
	No	3 (3.3)	5 (3.6)	8 (3.5)
	Not sure	84 (92.3)	99 (71.8)	183 (79.9)
SCH is a serious disease	Yes	22 (24.2)	64 (46.4)	86 (37.5)
	No	3 (3.3)	5 (3.6)	8 (3.5)
	Not sure	66 (72.5)	69 (50.0)	135 (59.0)
Willing to participate in mass treatment	Yes	89 (97.8)	135 (97.8)	224 (97.8)
	No	2 (2.2)	3 (2.2)	5 (2.2)
Attitude level	Positive	4 (4.4)	26 (18.8)	30 (13.1)
	Negative	87 (95.6)	112 (81.2)	199 (86.9)

### Practices towards prevention and control of intestinal schistosomiasis

Among 229 respondents who had awareness about SCH, 87.3% (200/229) avoided open defecation. However, only 10.5% (24/229) avoided swimming or bathing in water, while only 2.6% (6/229) avoided any contact with fresh water bodies. The majority of the respondents who had awareness about SCH avoided participation in agriculture (67.7%) and irrigation (81.2%). Overall, 43.2% of participants who ever heard about SCH had good prevention practices against SCH (Table 5).

**Table 5:** Practice of school-aged children and adults for schistosomiasis prevention and control in Amhara Regional State, northwest Ethiopia, February to June 2023

Practice	Response	Number (%)		
		SAC	Adult	Total
Open defecation	Yes	20 (22.0)	9 (6.5)	29 (12.7)
	No	71 (78.0)	129 (93.5)	200 (87.3)
Swimming/bathing in water	Yes	87 (95.6)	118 (85.5)	205 (89.5)
	No	4 (4.4)	20 (14.5)	24 (10.5)
Crossing surface water barefooted	Yes	25 (27.5)	72 (52.2)	97 (42.4)
	No	66 (72.5)	66 (47.8)	132 (57.6)
Playing near surface water	Yes	27 (29.7)	–	27 (29.7)
	No	64 (70.3)	–	64 (70.3)
Washing clothes in surface water	Yes	69 (75.8)	103 (74.6)	172 (75.1)
	No	22 (24.2)	35 (25.4)	57 (24.9)
Participation in agriculture	Yes	30 (33.0)	44 (31.9)	74 (32.3)
	No	61 (67.0)	94 (68.1)	155 (67.7)
Participation in irrigation	Yes	15 (16.5)	28 (20.3)	43 (18.8)
	No	76 (83.5)	110 (79.7)	186 (81.2)
Water source for drinking/washing	Piped	56 (61.5)	115 (83.3)	171 (74.7)
	Non-piped	35 (38.5)	23 (16.7)	58 (25.3)
Fetching surface water	Yes	41 (45.1)	40 (29.0)	81 (35.4)
	No	50 (54.9)	98 (71.0)	148 (64.6)
Participation in fishing	Yes	2 (2.2)	4 (2.9)	6 (2.6)
	No	89 (97.8)	134 (97.1)	223 (97.4)
Contact with freshwater bodies	Yes	88 (96.7)	135 (97.8)	223 (97.4)
	No	3 (3.3)	3 (2.2)	6 (2.6)
Having good practice	Yes	42 (46.2)	57 (41.3)	99 (43.2)
	No	49 (53.8)	81 (58.7)	130 (56.8)

## Discussion

In the present study, we have shown that the majority (80.8%) of respondents had never heard of SCH. The awareness level was higher in adults, males, and urban residents compared to their respective counterparts (p < 0.05). Only 7.9% and 13.1% of the respondents had good knowledge and a positive attitude towards SCH, respectively. The majority (89.5%) of respondents practiced swimming or bathing in freshwater, which predisposes them to the infection.

The social and behavioral change communication programs in Ethiopia primarily target SAC, and adolescents and adults are also considered in high transmission areas in SCH prevention and control [4]. In low and moderate transmission settings, educated children are expected to transfer information to their respective families; hence, all age groups of the community will have knowledge about etiology, transmission, risky practices, clinical signs and symptoms, treatment, and prevention methods. However, the results of the present study show that both SAC and people in the community have poor knowledge about intestinal SCH.

The limited number of respondents who have ever heard of SCH (19.2%) in the present study was in line with previous findings of 19.2% in Ethiopia [30] and 19.3% in Cameroon [31]. Compared to our findings, low levels of awareness were reported in Benishangul Gumuz Regional State, Ethiopia [22], Malawi [32] and Zimbabwe [33].The present finding was also lower than previous reports of 29.5% [25] and 97% in Ethiopia [23], 48% in Tanzania [34], 54% in South Africa [35], 69.7% in Gambia [36], 91.96% in Mozambique [37] and 96.2% in Gabon [38]. Variations in the health education programs, the study population, and the SCH transmission setting might contribute to the difference. For instance, a study from Benishangul Gumuz recruited ≥ 12-year-old participants [22] missing the important 6–11-year-old SAC who are primary targets for health education, hence the lower awareness level. Other studies from Ethiopia [23, 25] recruited participants from high-transmission settings, so the majority of them might experience the infection. In addition, four participants were recruited from each household in Tigray, Ethiopia; information transfer among family members might have inflated the awareness level [23].

Higher proportions of adult respondents were aware of SCH than SAC (24.7% vs.14.4%, p < 0.001). This might be due to the availability of diverse information sources for adults like community meetings, health institutions, a higher level of education, a relatively longer period of life experience and other social interactions, while SAC mainly get information from schools. This is supported by our study, where a higher proportion of adults mentioned that family or community (26.8% in adults vs. 17.6% in SAC) and health institutions or campaigns (18.8% in adults vs. 7.7% in SAC) were their sources of information. Recall bias in SC might also contribute to the lower awareness records than that of adults. More males were aware than females (p = 0.045) which is justifiable since males are more involved in social activities than females. Urban residents were more likely to access information sources compared to rural residents, as supported by the present study (p = 0.037).

Because the health education programs are cascaded by districts, there might be variation in implementation. This is supported by our study, which found a significant difference in awareness about SCH across districts (p < 0.001). The absence of a significant difference (p = 0.320) in awareness level among participants from low, moderate, and high SCH endemic districts shows limitations in designing and implementing interventions based on the local contexts of transmission intensity.

Among respondents who heard about SCH, only 10.9% knew that a worm is the etiologic agent for SCH. This finding is in line with reports of 11.25% in Tanzania [39], but it is lower as compared to 27% in Gabon [38] and 28% in Botswana [21]. Variation in the study population might make the difference. The two previous studies primarily targeted 6–13-year-old children. Differences in SCH endemicity might be another factor, as 36% of study participants from Gabon responded a history of SCH diagnosis [38], in contrast to 1.0% in the present study. Similarly, in our study, only 16 respondents knew the involvement of snails in the transmission of SCH, revealing the poor knowledge level about the etiology and the intermediate snail host in the study area.

In the present study, less than one-third (28.4%) of respondents who had awareness mentioned that swimming/bathing in freshwater is a risk factor for *Schistosoma* infection. This is higher than reports of 13.6% from Oromia, Ethiopia [40], but it is lower than reports of 43.9% in Tigray, Ethiopia [23], 85% in Botswana [21], and 88.6% in Benishangul Gumuz, Ethiopia [22]. The difference in knowledge about the risk factors might be due to the variation in the minimum age limit of respondents and the SCH endemicity of data collection districts across studies.

The majority (85.6%) of the respondents did not mention any of the signs and symptoms of SCH in the present study. This finding is similar to a previous systematic review report in SSA [41], but it is higher than findings of 28.9% in Tigray [23] and 79% in Bedeno, Ethiopia [40]. Abdominal pain (10.9%) was the most common manifestation mentioned by respondents. This corroborates the finding from Kenya [42]. Low knowledge about the signs and symptoms brings a challenge to drug-uptake [21]. Moreover, nearly half of our study participants did not know that SCH is a curable and preventable disease, similar to previous studies in Ethiopia [25], Mozambique [37], Yemen [43], and Kenya [42]. Not knowing that SCH is curable will affect the health seeking behavior of infected people, thereby hindering their participation in preventive activities and contributing to persistent parasite transmission.

Overall assessment of knowledge regarding SCH revealed that the proportion of respondents with good knowledge in the present study was very low (7.9%: 2.2% in SAC and 11.6% in adults). The FMoH has been implementing an integrated school-based awareness creation program for soil-transmitted helminths and SCH side-to-side with MDA campaigns. Despite these efforts, our study showed that SAC had a lower knowledge level compared to adults, which is an indication of poor implementation of school-based health education programs in the study area. Our finding is supported by previous reports in Ethiopia [30, 40, 44] and other SSA countries [34, 36]. A higher proportion of respondents (94.4%) with good knowledge were reported from Tigray region of Ethiopia because data were collected in an urban setting among respondents having frequent water contact. Moreover, four participants were selected in each household [23].

The very low positive attitude towards SCH in the present study (13.1%) corroborates with that of another study in Ethiopia [22], but it is contrary to findings in Uganda [28] and Gambia [36]. Variations in the study populations in terms of age, educational status, and access to health-related information, together with SCH endemicity, might contribute to the difference in attitude level. Differences in the cut-off point to declare a positive attitude might also be another factor attributing to the variation. Among respondents who heard of the disease, only 37.6% believed that SCH is a serious disease, which is lower than the study findings of 96% in Ethiopia [23] and 77.1% in Yemen [43]. Variations in the study population and incidence of SCH might make the difference. For instance, the study in Yemen recruited only SAC and the previous study in Ethiopia was done in a high transmission setting, unlike our study, which was conducted both from SAC and adults residing in low, moderate and high-transmission areas.

Assessment of control and prevention practices revealed that less than half (43.2%) of respondents who had awareness had good infection prevention practices against SCH which is lower than 57.7% reported from Gambia [36]. A majority (89.5%) of the respondents swam or bathed in freshwater, which is consistent with the previous finding of 88% in Ethiopia [23]. However, our finding is higher than what was previously reported in Yemen, where 58.8% practiced swimming or bathing [43]. Differences in access to safe water might contribute to this variation. For example, in our study, 25.3% of the respondents had no access to piped water. Similarly, 75.1% of respondents in our study practiced washing their clothes in surface water, which is in line with the previous finding of 74.3% in Yemen [43], but it is lower than the previous finding of 92.2% in Ethiopia [23]. Lack of access or frequent interruptions of piped water supply are possible justifications for the difference.

We believe the aim of the present study was achieved, as we have assessed the current status of KAP towards SCH both in school and community settings in the study area. However, this study was not without limitations. Social, cultural, and cognitive factors associated with KAP level were not explored. Recall bias, especially among children, might have affected the present result. Qualitative data was not collected, which would have generated more detailed data about the community’s perceptions or beliefs towards SCH.

## Conclusions

Intestinal schistosomiasis is endemic in the study area, and health education and promotion have been implemented for many years. Despite this, the majority of the residents still had poor knowledge, a negative attitude, and engaged in risky practices. Therefore, strengthening or increasing the KAP level of the studied population and others living in Amhara Regional State would greatly improve SCH prevention and control efforts. It would be advisable to support the school and health center education programs. We also recommend future qualitative studies aiming to assess reasons for low KAP levels and explore socio-cultural and cognitive factors associated with KAP towards SCH.

## Data Availability

All data generated or analyzed during this study are included in this published article.

## Abbreviations

FMoH: Federal Ministry of Health
KAP: Knowledge Attitude and Practice
MDA: Mass drug administration
SAC: School-aged children
SCH: Schistosomiasis
SSA: Sub-Saharan Africa
WASH: Water Sanitation and Hygiene
WHO: World Health Organization
