# Correction: Knowledge, attitude and practice towards intestinal schistosomiasis among school-aged children and adults in Amhara Regional State, northwest Ethiopia. A cross-sectional study

**DOI:** 10.1186/s41182-024-00648-7

**Published:** 2024-11-29

**Authors:** Getaneh Alemu, Endalkachew Nibret, Arancha Amor, Abaineh Munshea, Melaku Anegagrie

**Affiliations:** 1 Department of Medical Laboratory Science, Bahir Dar University, Bahir Dar, Ethiopia; 2 Biology Department, Science College, Bahir Dar University, Bahir Dar, Ethiopia; 3 Mundo Sano Foundation and Institute of Health Carlos III, Madrid, Spain; 4 Health Biotechnology Division, Institute of Biotechnology (IoB), Bahir Dar University, Bahir Dar, Ethiopia

**Correction: Tropical Medicine and Health (2024) 52:23** 10.1186/s41182-024-00584-6

Following publication of the original article [1], the authors reported that there was an error during percentage calculations in Tables 2 and 5.

The correct Tables 2 and 5 have been provided in this Correction. The corrections have been bolded.

The original article [1] has been corrected.

The incorrect Table 2 is:

**Table 2:** Knowledge about schistosomiasis among school-aged children and adults in Amhara Regional State, northwest Ethiopia, February to June 2023

Knowledge Area	Responses	Number (%)		
		SAC	Adults	Total
Ever heard about SCH or bilharzia (SAC = 634, adults = 558)	Yes	91 (14.4)	138 (24.7)	229 (19.2)
	No	543 (85.6)	420 (75.3)	963 (80.8)
Source of information for SCH (SAC = 91, adult = 138)	School	60 (65.9)	93 (67.4)	153 (66.8)
	Family/community	16 (17.6)	37 (26.8)	53 (23.1)
	Health institutions/campaigns	7 (7.7)	26 (18.8)	33 (14.4)
	Media (radio/TV)	12 (13.2)	9 (6.5)	21 (9.2)
Causative agent of SCH	Worm	5 (5.5)	20 (14.5)	25 (10.9)
	Bacteria/virus/mosquito	0 (0.0)	2 (1.4)	2 (0.9)
	Don’t know	86 (94.5)	116 (84.1)	202 (88.2)
Snails involvement in the transmission of SCH	Yes	1 (1.1)	15 (10.9)	16 (7.0)
	Don’t know	90 (98.9)	123 (89.1)	213 (93.0)
Activities attributed to SCH transmission	Defecating near fresh water	11 (12.1)	21 (15.2)	32 (14.0)
	Swimming/bathing or playing in fresh water	7 (7.7)	58 (42.0)	65 (28.4)
	Walking barefooted in water	1 (1.1)	10 (7.2)	11 (4.8)
	Don’t know	74 (81.3)	77 (55.8)	151 (66.0)
Means to know a person having SCH	By signs and symptoms	13 (14.3)	28 (20.3)	41 (17.9)
	By laboratory diagnosis	29 (31.9)	54 (39.1)	83 (36.2)
	Don’t know	61 (67.0)	82 (59.4)	143 (62.4)
Signs and symptoms of SCH	Abdominal pain	13 (14.3)	12 (8.7)	25 (10.9)
	Fever	1 (1.1)	4 (1.7)	5 (2.2)
	Bloody stool/diarrhea	–	5 (3.6)	5 (2.2)
	Nausea	–	3 (2.2)	3 (1.3)
	Cough	1 (1.1)	0 (0.0)	1 (0.4)
	Don’t know	78 (85.7)	118 (85.5)	196 (85.6)
SCH can be cured	Yes	50 (54.9)	84 (60.9)	134 (58.5)
	No	2 (2.2)	1 (0.7)	3 (1.3)
	Don’t know	39 (42.9)	53 (38.4)	92 (40.2)
Cure for SCH (SAC = 50, Adult = 84)	Modern medicine	50 (100.0)	83 (98.8)	133 (99.3)
	Traditional medicine	0 (0.0)	1 (1.2)	1 (0.7)
	Don’t know	0 (0.0)	1 (1.2)	1 (0.7)
SCH is preventable disease	Yes	40 (44.0)	83 (60.1)	123 (53.7)
	No	0 (0.0)	0 (0.0)	0 (0.0)
	Don’t know	51 (56.0)	55 (39.9)	106 (46.3)
Method of SCH prevention (SAC = 40, Adult = 83)	Avoid contact with freshwater bodies	4 (10.0)	4 (2.9)	8 (3.5)
	Use clean water for drinking and washing	14 (35.0)	54 (39.1)	68 (29.7)
	Participate in mass treatment	4 (10.0)	6 (4.3)	10 (4.4)
	Other	17 (42.5)	10 (7.2)	27 (11.8)
	Don’t know	7 (17.5)	8 (5.8)	15 (6.6)
Knowledge level	Good	2 (2.2)	16 (11.6)	18 (7.9)
	Poor	89 (97.8)	122 (88.4)	211 (92.1)

The correct Table 2 is:

**Table 2:** Knowledge about schistosomiasis among school-aged children and adults in Amhara Regional State, northwest Ethiopia, February to June 2023

Knowledge Area	Responses	Number (%)		
		SAC	Adults	Total
Ever heard about SCH or bilharzia (SAC = 634, adults = 558)	Yes	91(14.4)	138 (24.7)	229 (19.2)
	No	543 (85.6)	420 (75.3)	963 (80.8)
Source of information for SCH (SAC = 91, adult = 138)	School	60 (65.9)	93 (67.4)	153 (66.8)
	Family/community	16 (17.6)	37 (26.8)	53 (23.1)
	Health institutions/campaigns	7 (7.7)	26 (18.8)	33 (14.4)
	Media (radio/TV)	12 (13.2)	9 (6.5)	21 (9.2)
Causative agent of SCH	Worm	5 (5.5)	20 (14.5)	25 (10.9)
	Bacteria/virus/mosquito	0 (0.0)	2 (1.4)	2 (0.9)
	Don’t know	86 (94.5)	116 (84.1)	202 (88.2)
Snails involvement in the transmission of SCH	Yes	1 (1.1)	15 (10.9)	16 (7.0)
	Don’t know	90 (98.9)	123 (89.1)	213 (93.0)
Activities attributed to SCH transmission	Defecating near fresh water	11 (12.1)	21 (15.2)	32 (14.0)
	Swimming/bathing or playing in fresh water	7 (7.7)	58 (42.0)	65 (28.4)
	Walking barefooted in water	1 (1.1)	10 (7.2)	11 (4.8)
	Don’t know	74 (81.3)	77 (55.8)	**151 (65.9)**
Means to know a person having SCH	By signs and symptoms	13 (14.3)	28 (20.3)	41 (17.9)
	By laboratory diagnosis	29 (31.9)	54 (39.1)	83 (36.2)
	Don’t know	61 (67.0)	82 (59.4)	143 (62.4)
Signs and symptoms of SCH	Abdominal pain	13 (14.3)	12 (8.7)	25 (10.9)
	Fever	1 (1.1)	**4 (3.6)**	5 (2.2)
	Bloody stool/diarrhea	–	5 (3.6)	5 (2.2)
	Nausea	–	3 (2.2)	3 (1.3)
	Cough	1 (1.1)	0 (0.0)	1 (0.4)
	Don’t know	78 (85.7)	118 (85.5)	196 (85.6)
SCH can be cured	Yes	50 (54.9)	84 (60.9)	134 (58.5)
	No	2 (2.2)	1 (0.7)	3 (1.3)
	Don’t know	39 (42.9)	53 (38.4)	92 (40.2)
Cure for SCH (SAC = 50, Adult = 84)	Modern medicine	50 (100.0)	83 (98.8)	133 (99.3)
	Traditional medicine	0 (0.0)	1 (1.2)	1 (0.7)
	Don’t know	0 (0.0)	1 (1.2)	1 (0.7)
SCH is preventable disease **(SAC = 91, adult = 138)**	Yes	40 (44.0)	83 (60.1)	123 (53.7)
	No	0 (0.0)	0 (0.0)	0 (0.0)
	Don’t know	51 (56.0)	55 (39.9)	106 (46.3)
Method of SCH prevention (SAC = 40, Adult = 83)	Avoid contact with freshwater bodies	4 (10.0)	**4 (4.8)**	**8 (6.5)**
	Use clean water for drinking and washing	14 (35.0)	**54 (65.1)**	**68 (55.3)**
	Participate in mass treatment	4 (10.0)	**6 (7.2)**	**10 (8.1)**
	Other	17 (42.5)	**11 (13.3)**	**28 (22.8)**
	Don’t know	7 (17.5)	**8 (9.6)**	**15 (12.2)**
Knowledge level	Good	2 (2.2)	16 (11.6)	18 (7.9)
	Poor	89 (97.8)	122 (88.4)	211 (92.1)

The incorrect Table 5 is:

**Table 5:** Practice of school-aged children and adults for schistosomiasis prevention and control in Amhara Regional State, northwest Ethiopia, February to June 2023

Practice	Response	Number (%)		
		SAC	Adult	Total
Open defecation	Yes	20 (22.0)	9 (6.5)	29 (12.7)
	No	71 (78.0)	129 (93.5)	200 (87.3)
Swimming/bathing in water	Yes	87 (95.6)	118 (85.5)	205 (89.5)
	No	4 (4.4)	20 (14.5)	24 (10.5)
Crossing surface water barefooted	Yes	25 (27.5)	72 (52.2)	97 (42.4)
	No	66 (72.5)	66 (47.8)	132 (57.6)
Playing near surface water	Yes	27 (29.7)	–	27 (29.7)
	No	64 (70.3)	–	64 (70.3)
Washing clothes in surface water	Yes	69 (75.8)	103 (74.6)	172 (75.1)
	No	22 (24.2)	35 (25.4)	57 (24.9)
Participation in agriculture	Yes	30 (33.0)	44 (31.9)	74 (32.3)
	No	61 (67.0)	94 (68.1)	155 (67.7)
Participation in irrigation	Yes	15 (16.5)	28 (20.3)	43 (18.8)
	No	76 (83.5)	110 (79.7)	186 (81.2)
Water source for drinking/washing	Piped	56 (61.5)	115 (83.3)	171 (74.7)
	Non-piped	35 (38.5)	23 (16.7)	58 (25.3)
Fetching surface water	Yes	41 (45.1)	40 (29.0)	81 (35.4)
	No	50 (54.9)	98 (71.0)	148 (64.6)
Participation in fishing	Yes	2 (2.2)	4 (2.9)	6 (2.6)
	No	89 (97.8)	134 (97.1)	223 (97.4)
Contact with freshwater bodies	Yes	88 (96.7)	135 (97.8)	223 (97.4)
	No	3 (3.3)	3 (2.2)	6 (2.6)
Having good practice	Yes	42 (46.2)	57 (41.3)	99 (43.2)
	No	49 (53.8)	81 (58.6)	130 (56.8)

The correct Table 5 is:

**Table 5:** Practice of school-aged children and adults for schistosomiasis prevention and control in Amhara Regional State, northwest Ethiopia, February to June 2023

Practice	Response	Number (%)		
		SAC	Adult	Total
Open defecation	Yes	20 (22.0)	9 (6.5)	29 (12.7)
	No	71 (78.0)	129 (93.5)	200 (87.3)
Swimming/bathing in water	Yes	87 (95.6)	118 (85.5)	205 (89.5)
	No	4 (4.4)	20 (14.5)	24 (10.5)
Crossing surface water barefooted	Yes	25 (27.5)	72 (52.2)	97 (42.4)
	No	66 (72.5)	66 (47.8)	132 (57.6)
Playing near surface water	Yes	27 (29.7)	–	27 (29.7)
	No	64 (70.3)	–	64 (70.3)
Washing clothes in surface water	Yes	69 (75.8)	103 (74.6)	172 (75.1)
	No	22 (24.2)	35 (25.4)	57 (24.9)
Participation in agriculture	Yes	30 (33.0)	44 (31.9)	74 (32.3)
	No	61 (67.0)	94 (68.1)	155 (67.7)
Participation in irrigation	Yes	15 (16.5)	28 (20.3)	43 (18.8)
	No	76 (83.5)	110 (79.7)	186 (81.2)
Water source for drinking/washing	Piped	56 (61.5)	115 (83.3)	171 (74.7)
	Non-piped	35 (38.5)	23 (16.7)	58 (25.3)
Fetching surface water	Yes	41 (45.1)	40 (29.0)	81 (35.4)
	No	50 (54.9)	98 (71.0)	148 (64.6)
Participation in fishing	Yes	2 (2.2)	4 (2.9)	6 (2.6)
	No	89 (97.8)	134 (97.1)	223 (97.4)
Contact with freshwater bodies	Yes	88 (96.7)	135 (97.8)	223 (97.4)
	No	3 (3.3)	3 (2.2)	6 (2.6)
Having good practice	Yes	42 (46.2)	57 (41.3)	99 (43.2)
	No	49 (53.8)	**81 (58.7)**	130 (56.8)
